# Full-protein release assay in a reconstituted yeast translation system using GGQ-methylated eRF1

**DOI:** 10.3389/fmolb.2026.1933983

**Published:** 2026-10-02

**Authors:** Shuai Huang, Yichen Xu, Wenting Chen, Ziang Miao, Chang Liu, Riku Nagai, Muhoon Lee, Hitomi Seike, Shinji Nagata, Nono Takeuchi-Tomita

**Affiliations:** 1 Department of Computational Biology and Medical Sciences, Graduate School of Frontier Sciences, The University of Tokyo, Chiba, Japan; 2 Department of Integrated Biosciences, Graduate School of Frontier Sciences, The University of Tokyo, Chiba, Japan

**Keywords:** eRF1, GGQ methylation, peptide release, reconstituted yeast translation system, translation termination

## Abstract

Translation termination in eukaryotes requires the concerted action of release factors eRF1 and eRF3, together with accessory factors including eIF5A and Rli1, to achieve efficient peptide release from the ribosome. Here, we establish a yPURE system-based Termi-nLuc assay, in which pre-termination complexes (PreTCs), carrying full-length nanoluciferase (nLuc) peptidyl-tRNA, are formed by simply omitting release factors from the reconstituted yeast translation system, enabling rapid and sensitive analysis without the need for sucrose density gradient purification. To enable direct biochemical analysis of GGQ methylation—a physiologically important modification of eRF1 whose effects on peptide release activity had not previously been examined *in vitro*—we also established an *Escherichia coli* co-expression system of eRF1 and its GGQ-methylating enzyme, yielding fully GGQ-methylated eRF1. Using this platform, we characterized the individual and combined contributions of eRF1, eRF3, eIF5A, and Rli1 to peptide release, and demonstrated that the full complement of these factors is sufficient for efficient release. Under the current assay conditions, neither GGQ methylation of eRF1 nor the identity of the penultimate amino acid of the protein detectably affected peptide release activity, consistent with the possibility that eRF1•eRF3 recruitment to the ribosome, rather than the chemical hydrolysis step, is rate-limiting under these conditions. The yPURE-based Termi-nLuc assay is readily amenable to manipulation of the composition of translation factors, tRNAs, and mRNA sequences, making it a versatile and high-throughput-compatible platform for future studies of context-dependent regulation of translation termination, with potential applications in the screening of small molecules that modulate termination efficiency or promote readthrough of premature stop codons for the treatment of genetic diseases.

## 1 Introduction

The peptide release reaction of translation termination in eukaryotes is mediated by release factors eRF1 and eRF3, a ribosome-dependent GTPase. The eRF1•eRF3•GTP complex binds to the ribosomal A-site, thereby eRF1 recognizes the stop codon and mediates release of the nascent peptide from the P-site peptidyl-tRNA after GTP hydrolysis by eRF3 (Hellen, 2018). eRF1 carries a universally conserved GGQ motif that interacts with the peptidyltransferase center to promote peptidyl-tRNA hydrolysis, and the glutamine residue of this motif is subject to methylation by a dedicated methyltransferase. Although this modification has been shown to be biologically important (Heurgué-Hamard et al., 2005; Polevoda et al., 2006; Liu et al., 2009; Liu et al., 2010; Xu et al., 2024), its direct effect on peptide release activity has not been examined *in vitro*, as previous biochemical studies have relied on unmodified recombinant eRF1. The peptide release reaction is regulated by an orchestrated interplay among mRNA, rRNA, and release factors, as well as release factor-interacting proteins. It is further modulated by the length and sequence of the nascent peptide chain, ribosome-associated chaperones that contact nascent peptides, and other factors.

Defects in translation termination are associated with a broad spectrum of human diseases. Nonsense mutations, which introduce premature stop codons, account for more than 10% of all disease-causing mutations (Mort et al., 2008), and aberrant termination has been associated with a range of pathological conditions including neurological disorders and cancer. Understanding the molecular basis of context-dependent regulation of translation termination—including the roles of the nascent peptide sequence, mRNA context, and post-translational modifications of release factors such as eRF1 GGQ methylation—is therefore of broad biomedical significance. In particular, such knowledge is expected to inform the development of readthrough-inducing agents that suppress premature stop codons, a promising therapeutic strategy for genetic diseases caused by nonsense mutations. Furthermore, efficient and sensitive biochemical assays for studying eukaryotic termination mechanisms are essential for identifying and characterizing potential therapeutics that modulate translation termination.

Previous *in vitro* biochemical studies on translation termination have been based on short oligopeptide (4–6 amino acids) synthesis systems, where the length and sequence of the nascent peptide are limited (Schuller et al., 2017). Recently, an *in vitro* termination assay termed the “Termi-nLuc assay”, which assesses the release of full-length protein, has been developed (Susorov et al., 2020). In this system, nanoluciferase (nLuc) mRNA is translated in a cellular lysate in the presence of a catalytically inactive eRF1 mutant carrying a GGQ-to-AGQ mutation, which inhibits peptide release by endogenous eRF1 and promotes formation of a pre-termination complex (PreTC). PreTC is then purified by sucrose density gradient (SDG) fractionation and subjected to a peptide release reaction by exogenously added release factors. The tRNA-bound nLuc in PreTC is inactive because its C-terminal region must fold to form an active catalytic domain; thus, peptide release can be sensitively monitored by nLuc luminescence activity. Indeed, the effects of the C-terminal amino acid of the full-length protein on translation termination have been studied using the Termi-nLuc assay; the C-terminal proline has been shown to suppress release (Biziaev et al., 2025).

Our laboratory has established an *in vitro* reconstituted yeast protein synthesis system, the yPURE system, capable of synthesizing long polypeptides (Abe et al., 2020a; Nagai et al., 2021). The system utilizes CrPV IGR IRES (intergenic region internal ribosome entry site sequence from cricket paralysis virus)-containing mRNA, which drives translation initiation factor-independent internal ribosome entry, and a set of yeast elongation (eEF1A, eEF2, eEF3), termination (eRF1, eRF3), and recycling factors (Rli1, Hbs1, Dom34) (Abe et al., 2020a; Nagai et al., 2021). In the present study, we aimed to establish the Termi-nLuc assay applying the yPURE system (Figure 1). In this system, PreTC is formed by simply omitting release factors from the yPURE system, and can be subjected directly to the peptide release assay without SDG purification, which is, in the cellular lysate-based Termi-nLuc assay, a technically demanding and time-consuming process. Furthermore, we also established an *Escherichia coli* co-expression system of eRF1 and its GGQ-motif methyltransferase complex, which produces 100% GGQ-methylated eRF1. By combining these technical advances, we demonstrate that the yPURE-based Termi-nLuc assay provides a powerful platform for quantitative analysis of eukaryotic translation termination.

**FIGURE 1:**
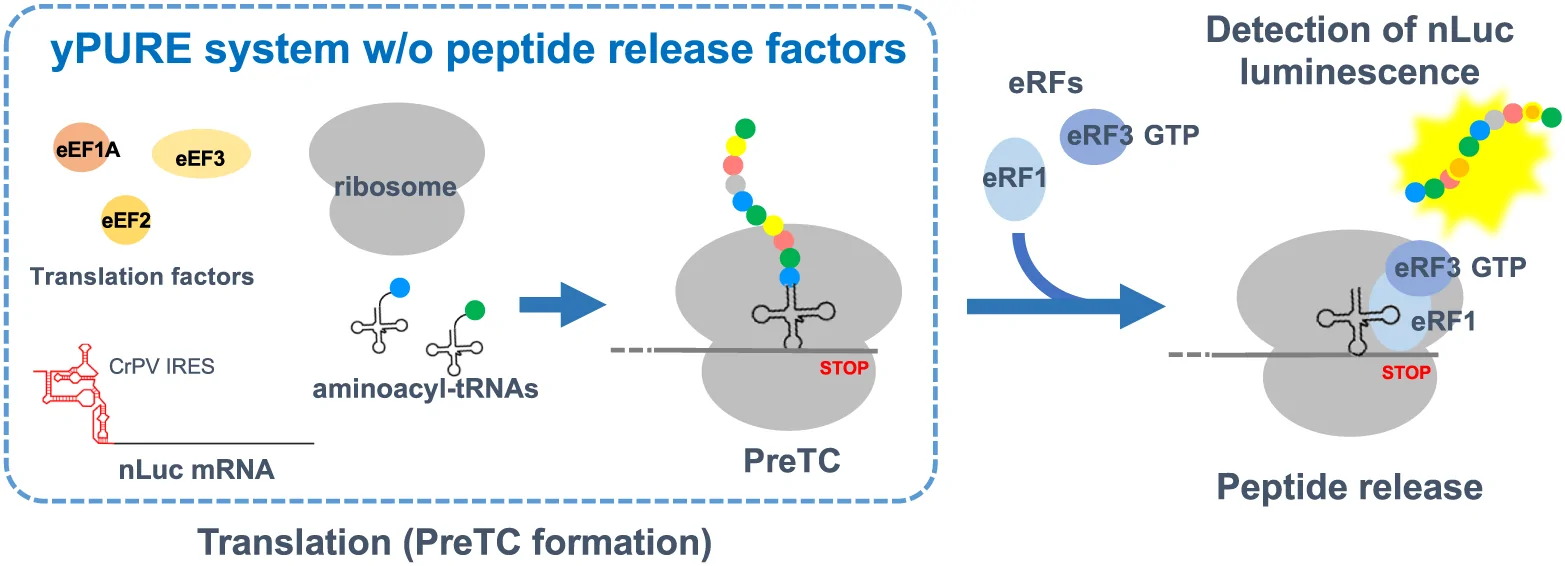
Schematic overview of the yPURE system-based Termi-nLuc assay. nLuc mRNA is translated in the yPURE system in the absence of release factors (eRFs), resulting in stable formation of a pre-termination complex (PreTC) carrying nLuc peptidyl-tRNA. The reaction mixture containing PreTC is then subjected directly to a peptide release reaction by the addition of eRFs, and peptide release is monitored by nLuc luminescence activity.

## 2 Materials and equipment

### 2.1 Proteins

Recombinant yeast eRF1 and GGQ-methylated eRF1 were expressed in *E*. *coli* and purified as described in the Methods section. Full-length eRF3 and two N-terminally truncated variants were used: eRF3Δ165, lacking the first 165 amino acid residues, is widely used in yeast translation studies (Schuller et al., 2017); and eRF3Δ248, lacking the first 248 amino acid residues, retains only the translational GTPase domain homologous to eEF1A. Both variants were expressed in *E. coli* and purified as described in the Methods section. Two forms of eIF5A were used: unmodified eIF5A (eIF5A_Lys) and hypusine-modified eIF5A (eIF5A_Hyp), both prepared as previously described (Nagai et al., 2021). Other components of the yPURE system, including ribosomes and translation elongation and recycling factors, were prepared as previously described (Abe et al., 2020a; Nagai et al., 2021).

### 2.2 mRNA constructs

Six mRNA constructs were used in this study (Table 1). All mRNAs contain the CrPV IGR IRES sequence and encode nanoluciferase (nLuc) as a reporter. The standard nLuc mRNA encodes full-length nLuc with a UAA stop codon. Five additional mRNA variants were designed to examine the effects of the penultimate codon on peptide release: four constructs encoding nLuc with a C-terminal proline residue specified by different Pro codons (CCA, CCU, CCC, and CCG), and one construct encoding nLuc with a C-terminal alanine residue (GCA). All mRNA constructs were prepared by *in vitro* transcription as previously described (Abe et al., 2020a; Nagai et al., 2021).

**TABLE 1:** Sequences of template DNAs for *in vitro* transcription of mRNAs used in this study.

IRES-FLAG-Pgk1-36-nLuc-HA
5′-GGGCCTAATACGACTCACTATAGGGAGACCGGAATTCAAAGCAAAAATGTGATCTTGCTTGTAAATACAATTTTGAGAGGTTAATAAATTACAAGTA GTGCTATTTTTGTATTTAGGTTAGCTATTTAGCTTTACGTTCCAGGATGCCTAGTGGCAGCCCCACAATATCCAGGAAGCCCTCTCTGC GGTTTTTCAGATTAGGTAGTCGAAAAACCTAAGAAATTTACCTgcaatggattacaaggacgacgacgacaagGAGCTCTCGTCGAAGCTCTCGGTGCAGGATCTCGAC CTCAAGGACAAGCGGGTGTTCATACGGGTGGACTTCAACGTGCCACTCGACGGTAAGAAGATAACGTCG **ACGGTGTTCACGCTCGAGGATTTCGTGG** **GTGATTGGCGGCAGACGGCAGGTTACAATCTCGATCAGGTGCTCGAGCAGGGTGGTGTGTCGTCGCTCTTTCAGAACCTCGGTGTGTCGGTGACGCCA** **ATACAGCGGATAGTGCTCTCGGGTGAGAACGGTCTCAAGATAGATATACATGTGATAATACCATACGAGGGTCTCTCGGGTGATCAGATGGGTCAGATAGAGA** **AGATATTCAAGGTGGTGTACCCAGTGGATGATCACCACTTTAAGGTGATACTCCACTACGGTACGCTCGTGATAGATGGTGTGACGCCAAACATGATAGATTA** **CTTTGGTCGGCCATACGAGGGTATAGCAGTGTTCGATGGTAAGAAGATAACGGTGACGGGTACGCTCTGGAACGGTAACAAGATAATAGACGAGCGGCTCAT**AAACC**CAGACGGTTCGCTCCTCTTCCGGGTGACGATAAACGGTGTGACGGGTTGGCGGCTCTGCGAGCGGATACTCGCA**tacccatacgacgtgccagactacgcaTAA--3′ Translation of ORF AMDYKDDDDKELSSKLSVQDLDLKDKRVFIRVDFNVPLDGKKITSTVFTLEDFVGDWRQTAGYNLDQVLEQGGVSSLFQNLGVSVTPIQRIVLSGENGLKIDIHVIIPYEGLSGDQMGQIEKIFKVVYPVDDHHFKVILHYGTLVIDGVTPNMIDYFGRPYEGIAVFDGKKITVTGTLWNGNKIIDERLINPDGSLLFRVTINGVTGWRLCERILAYPYDVPDYA

**IRES-FLAG-pgk1-36-nLuc-HA-Ala(GCA)-UGAC-polyA**
5′-GGGCCTAATACGACTCACTATAGGGAGACCGGAATTCAAAGCAAAAATGTGATCTTGCTTGTAAATACAATTTTGAGAGGTTAATAAATTACAA GTAGTGCTATTTTTGTATTTAGGTTAGCTATTTAGCTTTACGTTCCAGGATGCCTAGTGGCAGCCCCACAATATCCAGGAAGCCCTCTCTGCGGTTTTTCAGA TTAGGTAGTCGAAAAACCTAAGAAATTTACCTgcaatggattacaaggacgacgacgacaagGAGCTCTCGTCGAAGCTCTCGGTGCAGGATCTCGACCTCAAGGACAAGCG GGTGTTCATACGGGTGGACTTCAACGTGCCACTCGACGGTAAGAAGATAACGTCG **ACGGTGTTCACGCTCGAGGATTTCGTGGGTGATTGGCGGCAGACG** **GCAGGTTACAATCTCGATCAGGTGCTCGAGCAGGGTGGTGTGTCGTCGCTCTTTCAGAACCTCGGTGTGTCGGTGACGCCAATACAGCGGATAGTGCTCTC** **GGGTGAGAACGGTCTCAAGATAGATATACATGTGATAATACCATACGAGGGTCTCTCGGGTGATCAGATGGGTCAGATAGAGAAGATATTCA** **AGGTGGTGTACCCAGTGGATGATCACCACTTTAAGGTGATACTCCACTACGGTACGCTCGTGATAGATGGTGTGACGCCAAACATGATAGATTACTTTGG** **TCGGCCATACGAGGGTATAGCAGTGTTCGATGGTAAGAAGATAACGGTGACGGGTACGCTCTGGAACGGTAACAAGATAATAGACGAGCGGCTCATAAAC** **CCAGACGGTTCGCTCCTCTTCCGGGTGACGATAAACGGTGTGACGGGTTGGCGGCTCTGCGAGCGGATACTCGCA**tacccatacgacgtgccagactacgcaGCATGACAAAAAAAAAAAAAAAAAAAAAAAAAAAAAAAAAAAA-3′ Translation of ORF AMDYKDDDDKELSSKLSVQDLDLKDKRVFIRVDFNVPLDGKKITSTVFTLEDFVGDWRQTAGYNLDQVLEQGGVSSLFQNLGVSVTPIQRIVLSGENGLKIDIHVIIPYEGLSGDQMGQIEKIFKVVYPVDDHHFKVILHYGTLVIDGVTPNMIDYFGRPYEGIAVFDGKKITVTGTLWNGNKIIDERLINPDGSLLFRVTINGVTGWRLCERILAYPYDVPDYAA

IRES-FLAG-pgk1-36-nLuc-HA-Pro(CCA)-UGAC-polyA
5′-GGGCCTAATACGACTCACTATAGGGAGACCGGAATTCAAAGCAAAAATGTGATCTTGCTTGTAAATACAATTTTGAGAGGTTAATAAATTAC AAGTAGTGCTATTTTTGTATTTAGGTTAGCTATTTAGCTTTACGTTCCAGGATGCCTAGTGGCAGCCCCACAATATCCAGGAAGCCCTCTCTGCGGTTTTTCAG ATTAGGTAGTCGAAAAACCTAAGAAATTTACCTgcaatggattacaaggacgacgacgacaagGAGCTCTCGTCGAAGCTCTCGGTGCAGGATCTCGACCTCAAGGACAAGCG GGTGTTCATACGGGTGGACTTCAACGTGCCACTCGACGGTAAGAAGATAACGTCG **ACGGTGTTCACGCTCGAGGATTTCGTGGGTGATTGGCGGCAGACGG** **CAGGTTACAATCTCGATCAGGTGCTCGAGCAGGGTGGTGTGTCGTCGCTCTTTCAGAACCTCGGTGTGTCGGTGACGCCAATACAGCGGA** **TAGTGCTCTCGGGTGAGAACGGTCTCAAGATAGATATACATGTGATAATACCATACGAGGGTCTCTCGGGTGATCAGATGGGTCAGATAGAGAAGATATTCAAG** **GTGGTGTACCCAGTGGATGATCACCACTTTAAGGTGATACTCCACTACGGTACGCTCGTGATAGATGGTGTGACGCCAAACATGATAGATTACTTTGGTCGG** **CCATACGAGGGTATAGCAGTGTTCGATGGTAAGAAGATAACGGTGACGGGTACGCTCTGGAACGGTAACAAGATAATAGACGAGCGGCTCATAAACCC** **AGACGGTTCGCTCCTCTTCCGGGTGACGATAAACGGTGTGACGGGTTGGCGGCTCTGCGAGCGGATACTCGCA**tacccatacgacgtgccagactacgcaCCATGACAAAAAAAAAAAAAAAAAAAAAAAAAAAAAAAAAAAA-3′ Translation of ORF AMDYKDDDDKELSSKLSVQDLDLKDKRVFIRVDFNVPLDGKKITSTVFTLEDFVGDWRQTAGYNLDQVLEQGGVSSLFQNLGVSVTPIQRIVLSGENGLKIDIHVIIPYEGLSGDQMGQIEKIFKVVYPVDDHHFKVILHYGTLVIDGVTPNMIDYFGRPYEGIAVFDGKKITVTGTLWNGNKIIDERLINPDGSLLFRVTINGVTGWRLCERILAYPYDVPDYAP
5′-GGGCCTAATACGACTCACTATAGGGAGACCGGAATTCAAAGCAAAAATGTGATCTTGCTTGTAAATACAATTTTGAGAGGTTAATAAATT ACAAGTAGTGCTATTTTTGTATTTAGGTTAGCTATTTAGCTTTACGTTCCAGGATGCCTAGTGGCAGCCCCACAATATCCAGGAAGCCCTCTCTGCGGTTTT TCAGATTAGGTAGTCGAAAAACCTAAGAAATTTACCTgcaatggattacaaggacgacgacgacaagGAGCTCTCGTCGAAGCTCTCGGTGCAGGATCTCGACCTCAAGGA CAAGCGGGTGTTCATACGGGTGGACTTCAACGTGCCACTCGACGGTAAGAAGATAACGTCG **ACGGTGTTCACGCTCGAGGATTTCGTGGGTGATTGGCG** **GCAGACGGCAGGTTACAATCTCGATCAGGTGCTCGAGCAGGGTGGTGTGTCGTCGCTCTTTCAGAACCTCGGTGTGTCGGTGACGCCAATACAGCGGATA** **GTGCTCTCGGGTGAGAACGGTCTCAAGATAGATATACATGTGATAATACCATACGAGGGTCTCTCGGGTGATCAGATGGGTCAGATAGAGAAGATATTCAA** **GGTGGTGTACCCAGTGGATGATCACCACTTTAAGGTGATACTCCACTACGGTACGCTCGTGATAGATGGTGTGACGCCAAACATGATAGATTACTTTGGTC** **GGCCATACGAGGGTATAGCAGTGTTCGATGGTAAGAAGATAACGGTGACGGGTACGCTCTGGAACGGTAACAAGATAATAGACGAGCGGCTCATAAACCC** **AGACGGTTCGCTCCTCTTCCGGGTGACGATAAACGGTGTGACGGGTTGGCGGCTCTGCGAGCGGATACTCGCA**tacccatacgacgtgccagactacgcaCCTTGACAAAAAAAAAAAAAAAAAAAAAAAAAAAAAAAAAAAA-3′ Translation of ORF AMDYKDDDDKELSSKLSVQDLDLKDKRVFIRVDFNVPLDGKKITSTVFTLEDFVGDWRQTAGYNLDQVLEQGGVSSLFQNLGVSVTPIQRIVLSGENGLKIDIHVIIPYEGLSGDQMGQIEKIFKVVYPVDDHHFKVILHYGTLVIDGVTPNMIDYFGRPYEGIAVFDGKKITVTGTLWNGNKIIDERLINPDGSLLFRVTINGVTGWRLCERILAYPYDVPDYAP

IRES-FLAG-pgk1-36-nLuc-HA-Pro(CCC)-UGAC-polyA
5′-GGGCCTAATACGACTCACTATAGGGAGACCGGAATTCAAAGCAAAAATGTGATCTTGCTTGTAAATACAATTTTGAGAGGTTAATAAA TTACAAGTAGTGCTATTTTTGTATTTAGGTTAGCTATTTAGCTTTACGTTCCAGGATGCCTAGTGGCAGCCCCACAATATCCAGGAAGCCCTCTCTGCGGTTT TTCAGATTAGGTAGTCGAAAAACCTAAGAAATTTACCTgcaatggattacaaggacgacgacgacaagGAGCTCTCGTCGAAGCTCTCGGTGCAGGATCTCGACCTCAAGGA CAAGCGGGTGTTCATACGGGTGGACTTCAACGTGCCACTCGACGGTAAGAAGATAACGTCG **ACGGTGTTCACGCTCGAGGATTTCGTGGGTGATTGGCGG** **CAGACGGCAGGTTACAATCTCGATCAGGTGCTCGAGCAGGGTGGTGTGTCGTCGCTCTTTCAGAACCTCGGTGTGTCGGTGACGCCAATACAGCGGATA** **GTGCTCTCGGGTGAGAACGGTCTCAAGATAGATATACATGTGATAATACCATACGAGGGTCTCTCGGGTGATCAGATGGGTCAGATAGAGAAGATATTCA** **AGGTGGTGTACCCAGTGGATGATCACCACTTTAAGGTGATACTCCACTACGGTACGCTCGTGATAGATGGTGTGACGCCAAACATGATAGATTACTTTGGT** **CGGCCATACGAGGGTATAGCAGTGTTCGATGGTAAGAAGATAACGGTGACGGGTACGCTCTGGAACGGTAACAAGATAATAGACGAGCGGCTCATAAACC** **CAGACGGTTCGCTCCTCTTCCGGGTGACGATAAACGGTGTGACGGGTTGGCGGCTCTGCGAGCGGATACTCGCA**tacccatacgacgtgccagactacgcaCCCTGACAAAAAAAAAAAAAAAAAAAAAAAAAAAAAAAAAAAA-3′ Translation of ORF AMDYKDDDDKELSSKLSVQDLDLKDKRVFIRVDFNVPLDGKKITSTVFTLEDFVGDWRQTAGYNLDQVLEQGGVSSLFQNLGVSVTPIQRIVLSGENGLKIDIHVIIPYEGLSGDQMGQIEKIFKVVYPVDDHHFKVILHYGTLVIDGVTPNMIDYFGRPYEGIAVFDGKKITVTGTLWNGNKIIDERLINPDGSLLFRVTINGVTGWRLCERILAYPYDVPDYAP

IRES-FLAG-pgk1-36-nLuc-HA-Pro(CCG)-UGAC-polyA
5′-GGGCCTAATACGACTCACTATAGGGAGACCGGAATTCAAAGCAAAAATGTGATCTTGCTTGTAAATACAATTTTGAGAGGTTAA TAAATTACAAGTAGTGCTATTTTTGTATTTAGGTTAGCTATTTAGCTTTACGTTCCAGGATGCCTAGTGGCAGCCCCACAATATCCAGGAAGCCCTCTCTGC GGTTTTTCAGATTAGGTAGTCGAAAAACCTAAGAAATTTACCTgcaatggattacaaggacgacgacgacaagGAGCTCTCGTCGAAGCTCTCGGTGCAGGATCTCGACCT CAAGGACAAGCGGGTGTTCATACGGGTGGACTTCAACGTGCCACTCGACGGTAAGAAGATAACGTCG **ACGGTGTTCACGCTCGAGGATTTCGTGGGTGA** **TTGGCGGCAGACGGCAGGTTACAATCTCGATCAGGTGCTCGAGCAGGGTGGTGTGTCGTCGCTCTTTCAGAACCTCGGTGTGTCGGTGACGCC** **AATACAGCGGATAGTGCTCTCGGGTGAGAACGGTCTCAAGATAGATATACATGTGATAATACCATACGAGGGTCTCTCGGGTGATCAGATGGGTCAGATAG** **AGAAGATATTCAAGGTGGTGTACCCAGTGGATGATCACCACTTTAAGGTGATACTCCACTACGGTACGCTCGTGATAGATGGTGTGACGCCAAACATGATA** **GATTACTTTGGTCGGCCATACGAGGGTATAGCAGTGTTCGATGGTAAGAAGATAACGGTGACGGGTACGCTCTGGAACGGTAACAAGATAATAGACGAGC** **GGCTCATAAACCCAGACGGTTCGCTCCTCTTCCGGGTGACGATAAACGGTGTGACGGGTTGGCGGCTCTGCGAGCGGATACTCGCA**tacccatacgacgtgccagactacgcaCCGTGACAAAAAAAAAAAAAAAAAAAAAAAAAAAAAAAAAAAA-3′ Translation of ORF AMDYKDDDDKELSSKLSVQDLDLKDKRVFIRVDFNVPLDGKKITSTVFTLEDFVGDWRQTAGYNLDQVLEQGGVSSLFQNLGVSVTPIQRIVLSGENGLKIDIHVIIPYEGLSGDQMGQIEKIFKVVYPVDDHHFKVILHYGTLVIDGVTPNMIDYFGRPYEGIAVFDGKKITVTGTLWNGNKIIDERLINPDGSLLFRVTINGVTGWRLCERILAYPYDVPDYAP

Amino acid sequences of the translation products are also shown. Gray, T7 promoter; boxed G, transcription start site; single underline, CrPV IRES sequence; double underline, Pgk1-36 sequence; bold, nLuc sequence; lowercase letters, FLAG- or HA-tag sequence.

### 2.3 Reagents and equipment

Reagents and equipment used for the yPURE system-based translation reactions, the nLuc luminescence assay, and protein purification were as previously described (Nagai et al., 2021).

## 3 Methods

### 3.1 Preparation of GGQ-methylated eRF1 and eRF3 variants

#### 3.1.1 GGQ-methylated eRF1

GGQ methylation of eRF1 is catalyzed by the Mtq2–Trm112 methyltransferase complex, which uses the eRF1•eRF3•GTP ternary complex as its substrate (Heurgué-Hamard et al., 2005). To obtain fully GGQ-methylated eRF1, we constructed an *E. coli* co-expression vector in which eRF3Δ248, His6-tagged eRF1, Trm112, and Mtq2 were tandemly cloned into pET29b (eRF3Δ248_eRF1H6_Trm112_Mtq2/pET29b) (Figure 2A; Table 2). His6-tagged eRF1 was expressed in *E. coli* Rosetta(DE3)pLysS transformed with this plasmid and purified by affinity chromatography as previously described (Nagai et al., 2021).

**FIGURE 2:**
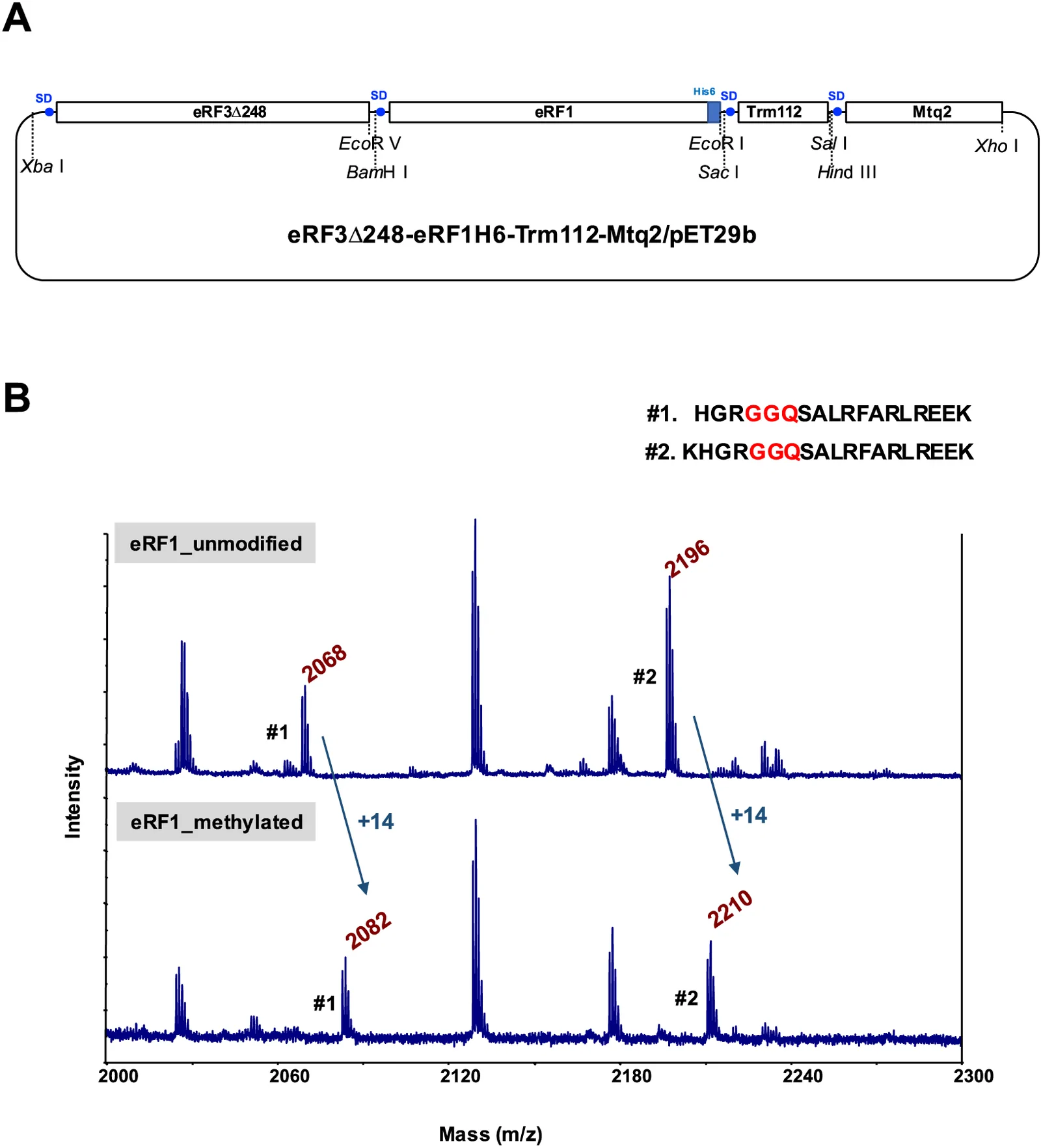
Preparation of GGQ-methylated eRF1. **(A)** Schematic representation of the co-expression vector eRF3Δ248_eRF1H6_Trm112_Mtq2/pET29b used for production of GGQ-methylated eRF1. The four genes are tandemly cloned under the control of a T7 promoter. Restriction enzyme sites used for cloning are indicated. SD, Shine-Dalgarno sequence; His6, hexahistidine tag. **(B)** MALDI-TOF mass spectrometry analysis of the GGQ motif-containing peptides (#1 and #2) derived from unmodified eRF1 (upper) and methylated eRF1 (lower) after lysyl endopeptidase digestion. An ion peak shift of +14 Da, corresponding to monomethylation, was observed for both peptides in the methylated sample, with no detectable peaks corresponding to the unmethylated forms, confirming nearly 100% methylation efficiency.

**TABLE 2:** Sequences of genes used in eRF3Δ248_eRF1H6_Trm112_Mtq2/pET29b.

eRF3Δ248 (optimized for *E. coli* expression)
5′-TCTAGAAATAATTTTGTTTAACTTTAAG **AAGGAG** ATATACATATGGAAGTGGTGAATGATATGTTCGGTGGTAAAGATCATGTGAGCCTGATTTTTATGGGTCATGTTGATGCAGGTAAAAGCACCATGGGTGGTAATCTGCTGTATCTGACCGGTAGCGTTGATAAACGTACCATCGAAAAATATGAACGCGAAGCAAAAGATGCAGGTCGTCAAGGTTGGTATCTGAGCTGGGTTATGGATACCAATAAAGAAGAACGTAACGACGGCAAAACCATTGAAGTTGGTAAAGCCTATTTCGAAACCGAGAAACGTCGTTATACCATTCTGGATGCACCGGGTCATAAAATGTATGTGAGCGAAATGATTGGTGGTGCAAGCCAGGCAGATGTTGGTGTTCTGGTTATTAGCGCACGTAAAGGTGAATATGAAACCGGCTTTGAACGTGGTGGTCAGACCCGTGAACATGCACTGCTGGCAAAAACCCAGGGTGTGAATAAAATGGTTGTGGTGGTTAACAAAATGGATGATCCGACCGTTAATTGGAGCAAAGAACGTTATGATCAGTGCGTTAGCAATGTGAGCAATTTTCTGCGTGCAATTGGCTACAACATTAAAACCGATGTTGTTTTTATGCCGGTGAGCGGTTATAGCGGTGCAAATCTGAAAGATCACGTTGATCCGAAAGAATGTCCGTGGTATACCGGTCCGACACTGCTGGAATATCTGGATACCATGAATCATGTGGATCGCCATATTAATGCACCGTTTATGCTGCCGATTGCAGCCAAAATGAAAGATCTGGGCACCATTGTTGAAGGCAAAATTGAAAGCGGTCATATCAAAAAGGGTCAGAGCACCCTGCTGATGCCGAATAAAACCGCAGTTGAAATTCAGAACATCTATAATGAGACAGAGAACGAAGTTGATATGGCCATGTGTGGTGAACAGGTTAAACTGCGTATTAAAGGTGTGGAAGAAGAAGATATTAGTCCGGGTTTTGTTCTGACCAGTCCGAAAAATCCGATTAAAAGCGTTACCAAATTTGTGGCCCAGATTGCAATTGTTGAACTGAAAAGCATTATTGCCGCAGGTTTTAGCTGTGTTATGCATGTGCATACCGCAATTGAAGAAGTGCATATTGTAAAACTGCTGCATAAACTGGAAAAAGGCACCAATCGTAAAAGCAAAAAGCCTCCGGCATTTGCAAAGAAAGGTATGAAAGTTATTGCCGTGCTGGAAACCGAAGCACCGGTTTGTGTTGAAACCTATCAGGATTATCCGCAGCTGGGTCGTTTTACCCTGCGTGATCAGGGCACCACCATTGCCATTGGTAAAATTGTTAAAATCGCCGAATAA-3′

eRF1H6 (optimized for *E. coli* expression)
5′-TCTAGAAATAATTTTGTTTAACTTTAAG **AAGGAG** ATATACATATGGATAACGAGGTGGAAAAGAACATCGAAATCTGGAAAGTTAAAAAGCTGGTTCAGAGCCTGGAAAAAGCACGTGGTAATGGCACCAGCATGATTAGCCTGGTTATTCCGCCTAAAGGTCAGATTCCGCTGTATCAGAAAATGCTGACCGATGAATATGGCACCGCAAGCAACATTAAAAGCCGTGTTAATCGTCTGAGCGTTCTGAGCGCAATTACCAGCACACAGCAGAAACTGAAACTGTATAATACCCTGCCGAAAAATGGTCTGGTTCTGTATTGTGGTGATATCATTACCGAAGATGGCAAAGAAAAGAAAGTGACCTTTGATATCGAACCGTACAAACCGATTAATACCAGCCTGTATCTGTGCGATAACAAATTTCATACCGAAGTGCTGAGCGAACTGCTGCAGGCAGATGATAAATTTGGTTTTATTGTGATGGATGGTCAGGGCACCCTGTTTGGTAGCGTTAGCGGTAATACCCGTACCGTTCTGCACAAATTTACCGTTGATCTGCCGAAGAAACATGGTCGTGGTGGTCAGAGCGCACTGCGTTTTGCACGTCTGCGTGAAGAAAAACGTCATAACTATGTTCGTAAAGTTGCCGAAGTTGCGGTGCAGAACTTTATTACCAATGATAAAGTGAACGTGAAGGGCCTGATTCTGGCAGGTAGCGCAGATTTCAAAACCGATCTGGCAAAAAGCGAACTGTTTGATCCGCGTCTGGCATGTAAAGTTATTAGCATTGTTGATGTGAGCTATGGTGGCGAAAATGGTTTTAATCAGGCAATTGAACTGAGTGCCGAAGCACTGGCAAATGTTAAATATGTGCAAGAGAAAAAGCTGCTGGAAGCCTATTTTGATGAAATTAGCCAGGATACCGGCAAATTTTGCTATGGTATTGATGATACCCTGAAAGCACTGGATCTGGGTGCAGTTGAAAAACTGATTGTGTTTGAAAACCTGGAAACCATCCGCTATACCTTTAAAGATGCGGAAGATAACGAAGTGATCAAATTTGCAGAACCGGAAGCCAAAGATAAAAGCTTTGCAATTGATAAAGCCACCGGTCAAGAAATGGATGTTGTTAGCGAAGAACCGCTGATTGAATGGCTGGCAGCAAACTATAAAAACTTTGGTGCAACCCTGGAATTCATCACCGATAAAAGCAGCGAAGGTGCACAGTTTGTTACCGGTTTTGGTGGTATTGGTGCAATGCTGCGTTACAAAGTTAATTTTGAACAGCTGGTCGATGAAAGCGAGGATGAATATTATGATGAAGATGAGGGCAGCGATTACGACTTTATCCACCACCACCACCACCACTAA-3′

Trm112 (optimized for *E. coli* expression)
5′-TCTAGAAATAATTTTGTTTAACTTTAAG **AAGGAG** ATATACATATGAAATTCCTGACCACCAACTTTCTGAAATGTAGCGTTAAAGCATGCGATACCAGCAATGATAATTTTCCGCTGCAGTATGATGGTAGCAAATGTCAGCTGGTTCAGGATGAAAGCATTGAATTTAATCCGGAATTCCTGCTGAATATTGTGGATCGTGTTGATTGGCCTGCAGTTCTGACCGTTGCAGCAGAACTGGGTAATAATGCACTGCCTCCGACCAAACCGAGCTTTCCGAGCAGCATTCAAGAACTGACCGATGATGATATGGCCATTCTGAATGATCTGCATACCCTGCTGCTGCAGACCAGCATTGCCGAAGGTGAAATGAAATGTCGTAATTGCGGTCACATCTACTACATCAAAAATGGCATTCCGAATCTGCTGTTACCGCCTCATCTGGTTTAA-3′

Mtq2 (optimized for *E. coli* expression)
5′-TCTAGAAATAATTTTGTTTAACTTTAAG **AAGGAG** ATATACATATGCTGCCGACACCGTATGTTAAATGCGATTATGATAAAGTGTATGAGCCTGCCGAAGATAGCTTTCTGATTCTGGATTGTCTGGAAAAAGAGCACGATTTTCTGAAACAGAAATTTGGTAATCGTCTGGCCATTGTGTGTGAAATTGGTAGCGGTAGTGGTATTGTTACCACCTTTCTGATGCAGAACAAAATCATTCCGCAAGAAAACAGCATTCATCTGGCCGTTGATATTAATCCGTGGGCATTAGAAGCAACCCTGGATACCGCAAAACTGAATAGCTGTAAAAGCAGCTTTCTGGAAGTTATTCAGGCAGATCTGAATAGCAGCATTCGTAATAATCAGGTTGATGTGCTGATTTTCAACCCTCCGTATGTTCCGGCAGAATGTGTTCCGGATGTTCCTGGTAGCCGTGAAGAGGCAGATCAGTGGCTGGATCTGGCACTGTTAGGTGGTAAAGATGGTATGGCAATTACCGATAAACTGCTGCGTCAGCTGGAACAAATTCTGAGTCCGGATGGTGTTGCATATATTCTGTTTTGTGCACGCAACAAACCGAAAGAAGTGATTAAACGTTTCGTGGACACCTACAAATGGAACGTGAAACTGATTGAAACCCGTAAAGCAGGTTGGGAAGTTCTGAGCGTTTATAGCTTTACCCGTTAA-3′

Each gene was cloned into pET29b using the restriction sites indicated in Figure 2A. Single underline, leader sequence including SD sequence (bold); double underline, hexahistidine tag; boxed, start/stop codon.

The methylation efficiency of the purified eRF1 was assessed by MALDI-TOF mass spectrometry. Briefly, the protein was reduced with DTT at 50 °C for 30 min and alkylated with iodoacetamide at room temperature for 90 min in the dark. The reduced and alkylated protein was digested with lysyl endopeptidase (1 μg) at 37 °C for 2 h, and the resulting peptides were desalted using a ZipTip C18 cartridge prior to analysis. Samples were mixed 1:1 with saturated α-cyanohydroxycinnamic acid in 60% acetonitrile and analyzed using a MALDI-TOF MS instrument (AB Sciex 5800 System) in a positive-ion reflector mode with an accelerating voltage of 30 kV. This analysis confirmed that eRF1 was methylated at nearly 100% efficiency (Figure 2B).

#### 3.1.2 eRF3 variants

Three eRF3 variants were used in this study. The expression vector for C-terminal histidine-tagged eRF3-Full-length (eRF3-Full-length/pET21a) was constructed as previously described (Kurata et al., 2013). The expression vector for C-terminal histidine-tagged eRF3Δ165 (eRF3Δ165/pET21a) was constructed as previously described (Abe et al., 2020a; Nagai et al., 2021). The expression vector for C-terminal histidine-tagged eRF3Δ248 (eRF3Δ248/pET21a) was constructed in this study (Table 3). All three eRF3 variants were expressed in *E. coli* BL21(DE3) and purified as previously described (Nagai et al., 2021).

**TABLE 3:** Sequence of the eRF3Δ248 gene used in eRF3Δ248/pET21a.

eRF3Δ248
5'-GAAGTTGTTAACGATATGTTTGGTGGTAAAGATCACGTTTCTTTAATTTTCATGGGTCATGTTGATGCCGGTAAATCTACTATGGGTGGTAATCTACTATACTTGACTGGCTCTGTGGATAAGAGAACTATTGAGAAATATGAAAGAGAAGCCAAGGATGCAGGCAGACAAGGTTGGTACTTGTCATGGGTCATGGATACCAACAAAGAAGAAAGAAATGATGGTAAGACTATCGAAGTTGGTAAGGCCTACTTTGAAACTGAAAAAAGGCGTTATACCATATTGGATGCTCCTGGTCATAAAATGTACGTTTCCGAGATGATCGGTGGTGCTTCTCAAGCTGATGTTGGTGTTTTGGTCATTTCCGCCAGAAAGGGTGAGTACGAAACCGGTTTTGAGAGAGGTGGTCAAACTCGTGAACACGCCCTATTGGCCAAGACCCAAGGTGTTAATAAGATGGTTGTCGTCGTAAATAAGATGGATGACCCAACCGTTAACTGGTCTAAGGAACGTTACGACCAATGTGTGAGTAATGTCAGCAATTTCTTGAGAGCAATTGGTTACAACATTAAGACAGACGTTGTATTTATGCCAGTATCCGGCTACAGTGGTGCAAATTTGAAAGATCACGTAGATCCAAAAGAATGCCCATGGTACACCGGCCCAACTCTGTTAGAATATCTGGATACAATGAACCACGTCGACCGTCACATCAATGCTCCATTCATGTTGCCTATTGCCGCTAAGATGAAGGATCTAGGTACCATCGTTGAAGGTAAAATTGAATCCGGTCATATCAAAAAGGGTCAATCCACCCTACTGATGCCTAACAAAACCGCTGTGGAAATTCAAAATATTTACAACGAAACTGAAAATGAAGTTGATATGGCTATGTGTGGTGAGCAAGTTAAACTAAGAATCAAAGGTGTTGAAGAAGAAGACATTTCACCAGGTTTTGTACTAACATCGCCAAAGAACCCTATCAAGAGTGTTACCAAGTTTGTAGCTCAAATTGCTATTGTAGAATTAAAATCTATCATAGCAGCCGGTTTTTCATGTGTTATGCATGTTCATACAGCAATTGAAGAGGTACATATTGTTAAGTTATTGCACAAATTAGAAAAGGGTACCAACCGTAAGTCAAAGAAACCACCTGCTTTTGCTAAGAAGGGTATGAAGGTCATCGCTGTTTTAGAAACTGAAGCTCCAGTTTGTGTGGAAACTTACCAAGATTACCCTCAATTAGGTAGATTCACTTTGAGAGATCAAGGTACCACAATAGCAATTGGTAAAATTGTTAAAATTGCCGAG-3′

The DNA fragment encoding eRF3Δ248, corresponding to residues 249–685 of *S. cerevisiae* eRF3 (YDR172W, SGD ID: S000002579), was cloned into pET21a using the *Nde* I and *Xho* I sites.

### 3.2 Translation in the yPURE system

Unless otherwise specified, standard translation reactions in the yPURE system were performed as described previously (Nagai et al., 2021), with slight modifications: eEF1A and eEF2 were used at final concentrations of 10.0 µM and 0.1 µM, respectively. The standard translation mixtures (20 µL) contained 50 mM HEPES-KOH (pH 7.5), 100 mM potassium glutamate, 5 mM Mg(OAc)_2_, 0.25 mM spermidine, 1 mM DTT, 0.15 mM of each amino acid (except methionine and cysteine), 0.05 mM methionine, 0.1 mM cysteine, 1 mM ATP, 1 mM GTP, 20 mM creatine phosphate, 100 nM creatine kinase, 20 nM myokinase, 15 nM nucleoside-diphosphate kinase, 15 nM pyrophosphatase, 10 µM eEF1A, 0.1 µM eEF2, 0.5 µM eEF3, 0.5 µM unmodified eRF1, 0.5 µM eRF3Δ165, 0.5 µM Hbs1, 0.5 µM Dom34, 0.125 µM Rli1, 0.5 µM 80S ribosome, and 0.125 A_260_ units of aminoacyl-tRNA mixture, and indicated concentration of mRNA. The reaction mixture was incubated at 30 °C for the indicated time.

To monitor nLuc luminescence activity in standard translation reactions, a NanoLuciferase assay was performed using a Nano-Glo Luciferase Assay System (Promega) as described in Nagai et al. (2021). Briefly, the 2 µL reaction was stopped by adding 18 µL of stop solution (20 mM HEPES-KOH [pH 7.5], 100 mM KOAc, 2 mM Mg(OAc)2, 0.1 mg/mL RNase A). RNase A was omitted from the stop solution when nLuc luminescence activity was measured without RNase A treatment. The mixture was combined with 0.2 µL of the substrate in a white 96-well half-area plate. After incubation for 17 min at room temperature, luminescence was detected using a GloMax Multi Detection System (Promega). Using this assay, we confirmed that translation reactions performed in the presence or absence of release factors, with or without RNase A treatment, allow stable formation of the pre-termination complex (PreTC) to be monitored (Figure 3).

**FIGURE 3:**
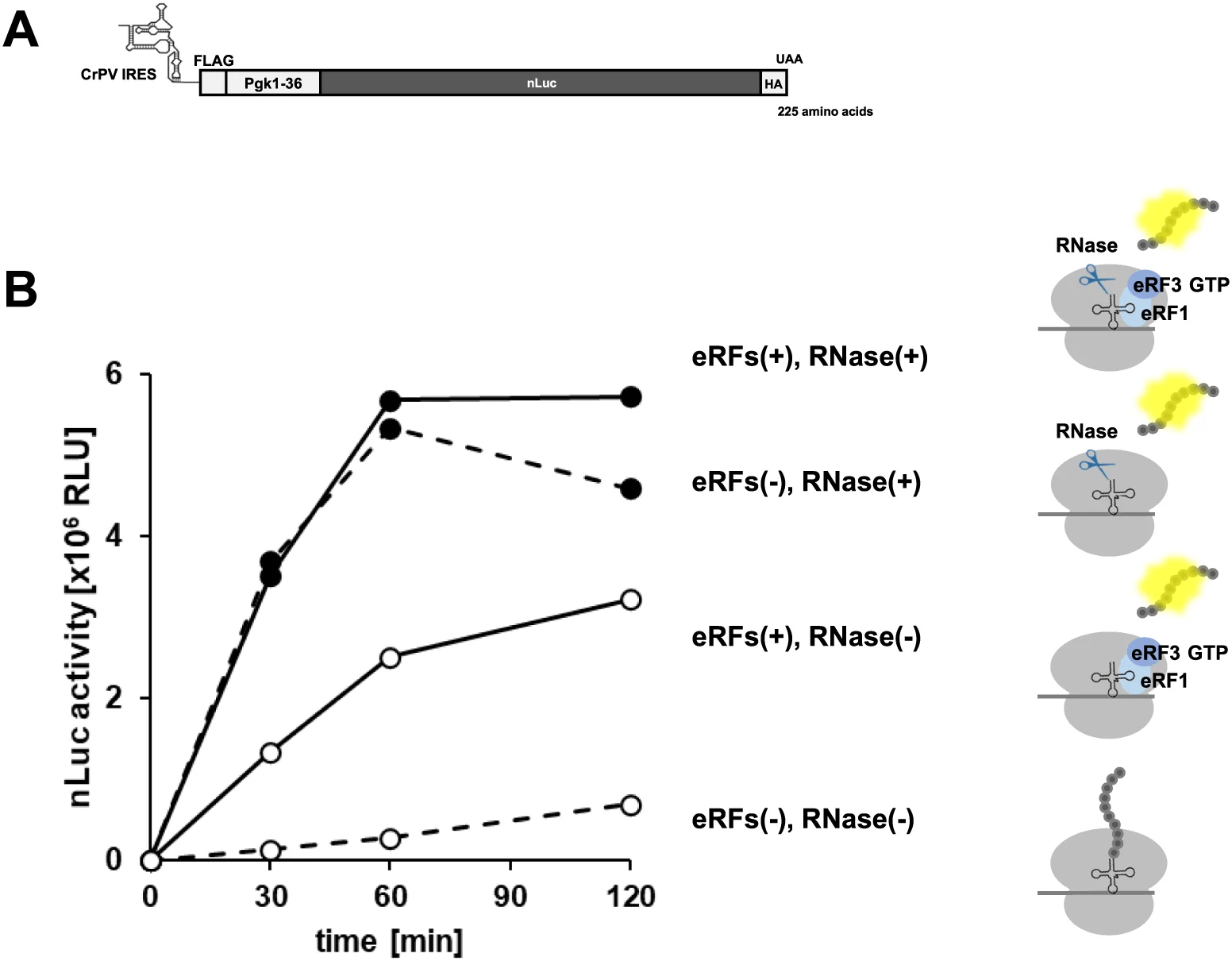
Formation of nLuc pre-termination complex (PreTC) in the yPURE system. **(A)** Schematic representation of the nLuc mRNA construct. The mRNA contains the CrPV IGR IRES sequence at the 5′end, followed by a FLAG tag, the Pgk1 N-terminal 36 amino acid sequence, the nLuc coding sequence, an HA tag, and a UAA stop codon (225 amino acids in total). **(B)** nLuc mRNA was translated in the yPURE system in the presence or absence of release factors (eRFs; eRF1 and eRF3) for the indicated times. nLuc luminescence activity was measured with (RNase(+)) or without (RNase(−)) RNase A treatment. Schematic representations of the ribosomal complexes under each condition are shown on the right. The representative data are presented.

### 3.3 Formation of pre-termination complex (PreTC) using the yPURE system

To form pre-termination complexes (PreTCs), translation reactions were performed as described in Section 3.2, except that release and recycling factors (eRF1, eRF3, Rli1, Dom34, and Hbs1) were omitted. Unless otherwise specified, 0.5 µM mRNA was used. The reaction mixture was incubated at 30 °C for 30 min. The total amount of PreTC formed in each reaction was quantified by treating translation products with RNase A, which releases nLuc from all peptidyl-tRNAs, and measuring the resulting nLuc luminescence activity (see Section 3.4).

### 3.4 Peptide release assay

PreTC formed in Section 3.3 was immediately used for the peptide release assay. The 2 µL translation reaction containing PreTC was transferred into 18 µL of the indicated solution in a white 96-well half-area plate and incubated for the indicated time at 30 °C. Three types of solutions were used: PreTC Buffer (20 mM HEPES-KOH [pH 7.5], 100 mM K-Glu [pH 7.5], 5 mM Mg(OAc)_2_, 0.25 mM spermidine, 1 mM DTT, 0.16 mM GTP, 0.32 mM ATP), RNase solution (20 mM HEPES-KOH [pH 7.5], 100 mM KOAc [pH 7.5], 2 mM Mg(OAc)_2_, 1 mM DTT, 0.1 mg/mL RNase A), and Release Factor solution (PreTC Buffer supplemented with the indicated amounts of release factors). Following incubation, 0.2 µL of nanoluciferase substrate (Promega) was added to each sample, and after a further 17 min incubation at 30 °C, luminescence was detected using a GloMax Multi Detection System (Promega).

The relative release activity (%) was calculated using the following formula: relative release activity (%) = (nLuc activity with release factors − nLuc activity with PreTC buffer)/(nLuc activity with RNase A− nLuc activity with PreTC buffer) × 100.

### 3.5 Analysis of release factor activities

To characterize the peptide release activity of each release factor, their effects were analyzed in a stepwise manner using nLuc-PreTC. The amounts of release factors are expressed as multiples (×) of the total 80S ribosome concentration (0.05 µM) present in the peptide release reaction; for example, 1× corresponds to 0.05 µM. Unless otherwise specified, 0.5 µM mRNA was used for PreTC formation in this section.

First, the effect of eRF1 alone on peptide release was examined. Methylated eRF1 was added at final concentrations of 0.05×, 0.25×, and 1× to nLuc-PreTC and incubated at 30 °C for up to 120 min eRF1 alone induced peptide release in a concentration-dependent manner, with negligible release at 0.05× (Figure 4A).

**FIGURE 4:**
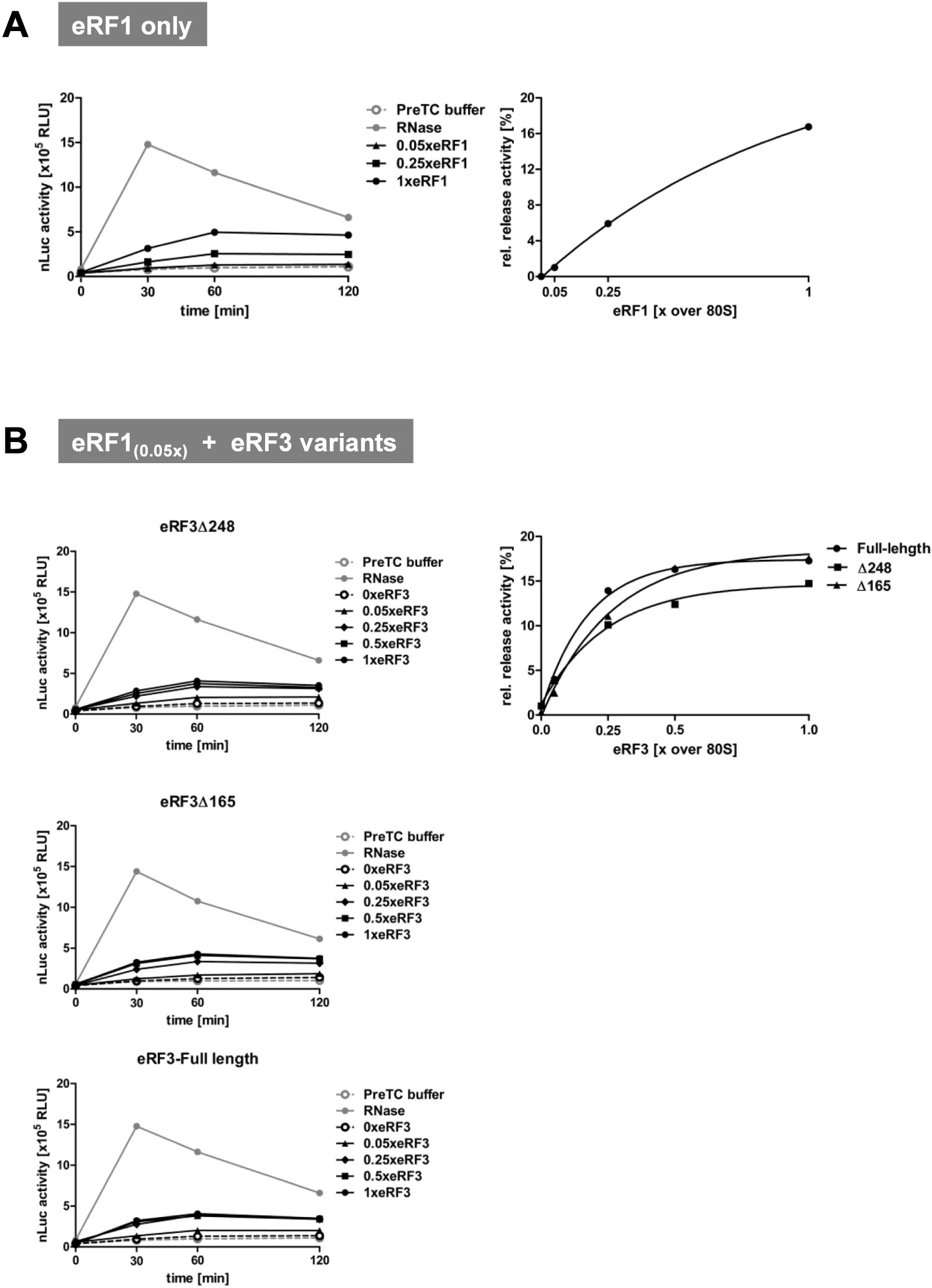
Concentration-dependent peptide release activities of eRF1 and eRF3. **(A)** Peptide release activity of methylated eRF1 alone. nLuc-PreTC was incubated with methylated eRF1 at the indicated concentrations (0.05×, 0.25×, and 1× relative to 80S ribosomes) at 30 °C for up to 120 min. Left: time course of nLuc luminescence activity. Right: relative release activity at 30 min as a function of eRF1 concentration. **(B)** Stimulatory effect of eRF3 variants on eRF1-mediated peptide release. nLuc-PreTC was incubated with methylated eRF1 (0.05×) and increasing amounts of eRF3Δ248 (top left), eRF3Δ165 (middle left), or eRF3-Full-length (bottom left) at 30 °C for up to 120 min. Right: relative release activity at 30 min as a function of eRF3 concentration for all three variants. The representative data are presented.

Next, the stimulatory effect of eRF3 on eRF1-mediated peptide release was examined. nLuc-PreTC was incubated with a fixed amount of methylated eRF1 (0.05×) and increasing amounts of eRF3 (0, 0.05, 0.25, 0.5, and 1×) at 30 °C for up to 120 min. All three eRF3 variants (eRF3-Full-length, eRF3Δ165, and eRF3Δ248) stimulated eRF1-mediated peptide release in a concentration-dependent manner, reaching comparable maximal release activities at saturating amounts (>0.5× eRF3) (Figure 4B).

The effect of eIF5A on peptide release was then examined. nLuc-PreTC was incubated with methylated eRF1 (0.05×) and eRF3Δ248 (0.5×), in the presence or absence of eIF5A_Lys or eIF5A_Hyp (1×) at 30 °C for up to 60 min. Both forms of eIF5A stimulated peptide release under both conditions, and hypusine-modified eIF5A (eIF5A_Hyp) showed higher stimulatory activity than unmodified eIF5A (eIF5A_Lys) (Figure 5).

**FIGURE 5:**
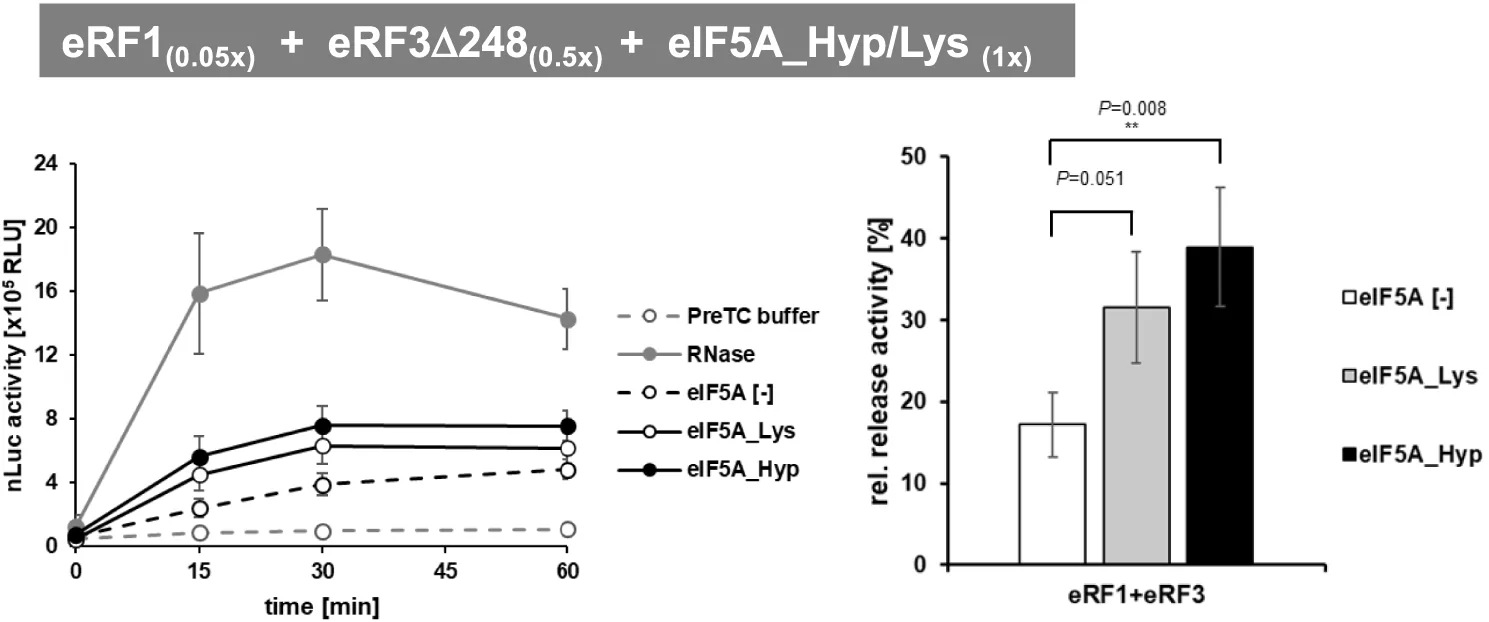
Stimulatory effect of eIF5A on peptide release. nLuc-PreTC was incubated with methylated eRF1 (0.05×) and eRF3Δ248 (0.5×) in the presence or absence of eIF5A_Lys or eIF5A_Hyp (1×) at 30 °C for up to 60 min. Left: time course of nLuc luminescence activity. Right: relative release activity at 30 min in the presence of eRF1 (0.05×) and eRF3Δ248 (0.5×) with the indicated forms of eIF5A. Data are presented as mean ± SD (n = 3). P values were calculated by two-tailed Welch’s t-test (**P < 0.01).

Finally, the effect of Rli1 on peptide release was examined. nLuc-PreTC was incubated with methylated eRF1 (0.05×), eRF3Δ248 (0.5×), and eIF5A_Hyp (1×) in the presence of increasing amounts of Rli1 (0, 0.05, 0.25, 1, and 2.5×) at 30 °C for up to 120 min. Addition of Rli1 further stimulated peptide release in a concentration-dependent manner (Figure 6).

**FIGURE 6:**
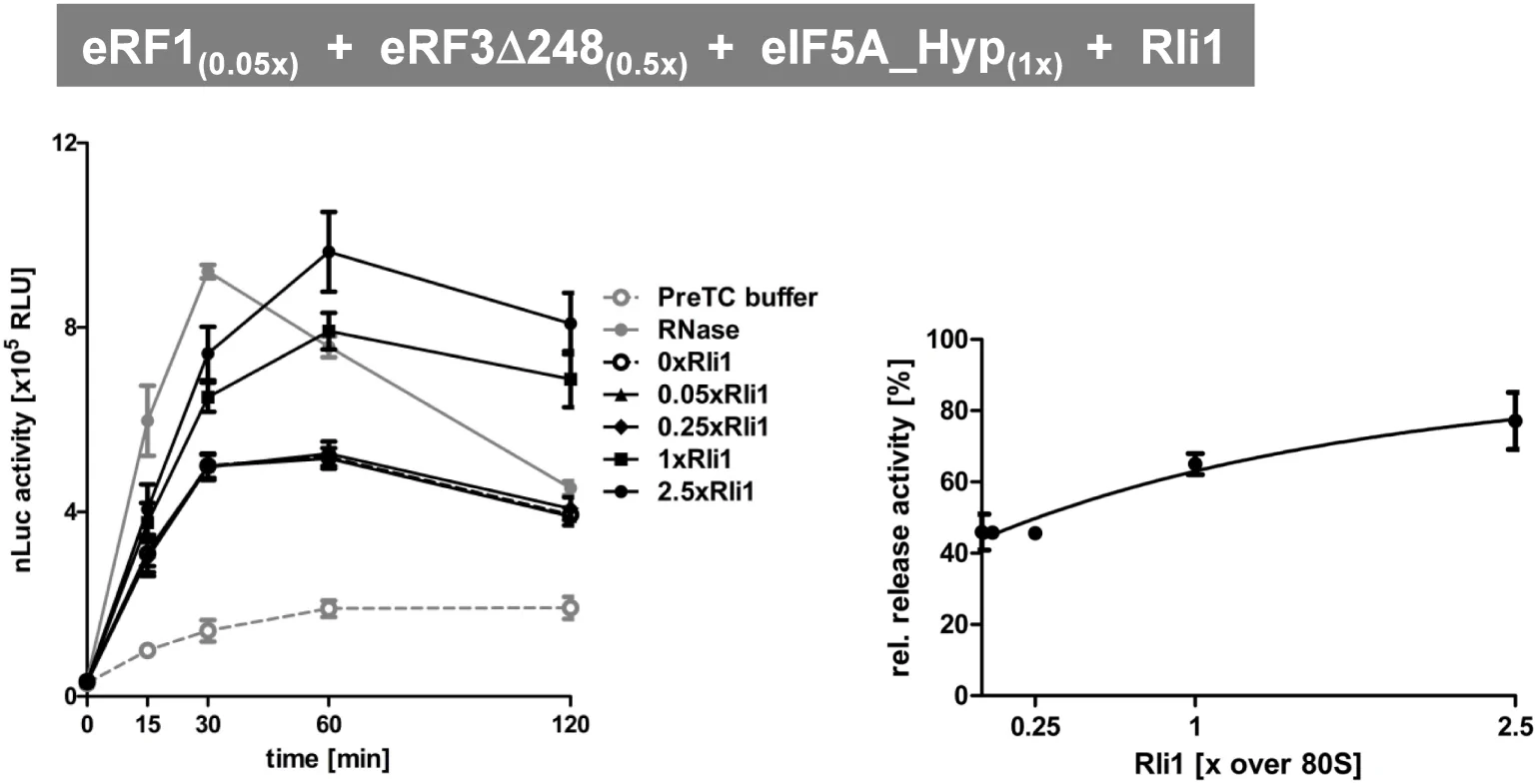
Stimulatory effect of Rli1 on peptide release. nLuc-PreTC was incubated with methylated eRF1 (0.05×), eRF3Δ248 (0.5×), and eIF5A_Hyp (1×) in the presence of increasing amounts of Rli1 (0, 0.05, 0.25, 1, and 2.5× relative to 80S ribosomes) at 30 °C for up to 120 min. Left: time course of nLuc luminescence activity. Right: relative release activity at 30 min as a function of Rli1 concentration. Data are presented as mean ± SD (n = 3).

### 3.6 Context-dependent analysis of translation termination

To examine the effect of the penultimate amino acid on peptide release, PreTCs were formed using five nLuc mRNA variants (Figure 7A; Table 1) as described in Section 3.3, with 5 µM mRNA. The mRNA constructs encode nLuc with a C-terminal alanine residue specified by GCA, or a C-terminal proline residue specified by one of four Pro codons (CCA, CCU, CCC, or CCG), followed by a stop codon.

**FIGURE 7:**
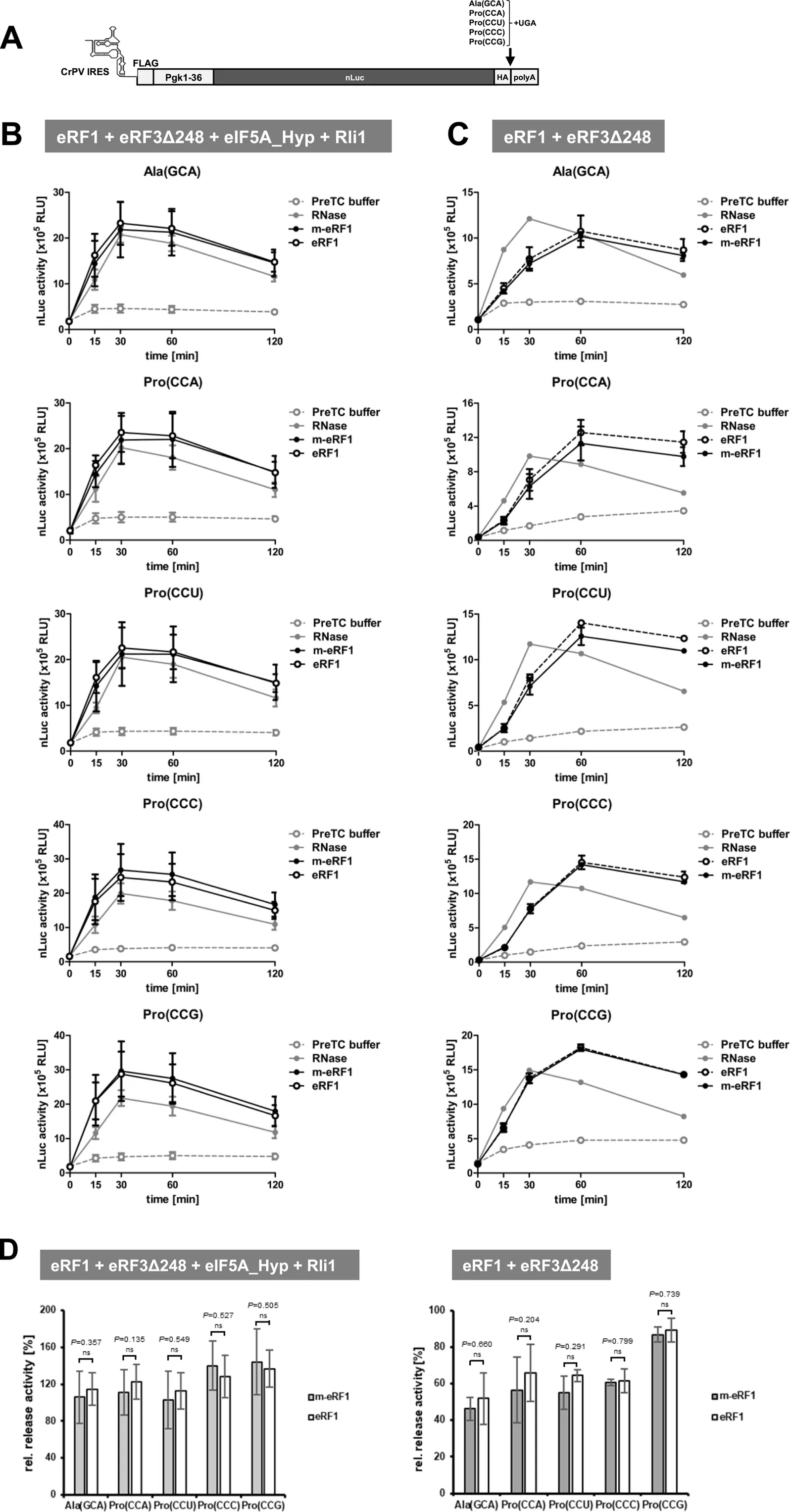
Context-dependent analysis of translation termination. **(A)** Schematic representation of the nLuc mRNA variants used for context-dependent analysis. Five mRNA variants were designed with different penultimate codons inserted immediately before the UGA stop codon: Ala(GCA), Pro(CCA), Pro(CCU), Pro(CCC), and Pro(CCG). All constructs carry a 3′-terminal poly **(A)** 36 sequence. **(B)** Peptide release reactions under the full release factor condition. nLuc-PreTCs carrying the indicated C-terminal residues were incubated with unmodified eRF1 (eRF1) or methylated eRF1 (m-eRF1) (0.05×), eRF3Δ248 (0.5×), eIF5A_Hyp (1×), and Rli1 (1×) at 30 °C for up to 120 min. Time courses of nLuc luminescence activity for each construct are shown. **(C)** Peptide release reactions under the minimal condition. nLuc-PreTCs were incubated with unmodified eRF1 (eRF1) or methylated eRF1 (m-eRF1) (0.05×) and eRF3Δ248 (0.5×) alone at 30 °C for up to 120 min. Time courses of nLuc luminescence activity for each construct are shown. **(D)** Relative release activity at 30 min under the full release factor condition (left, corresponding to **B**) and the minimal condition (right, corresponding to **C**) for each construct. Data are presented as mean ± SD (n = 3). P values were calculated by two-tailed Welch’s t-test (ns, not significant).

Peptide release reactions were performed as described in Section 3.4 under two conditions: a full release factor condition using unmodified eRF1 or methylated eRF1 (0.05×), eRF3Δ248 (0.5×), eIF5A_Hyp (1×), and Rli1 (1×) (Figure 7B), and a minimal condition using unmodified eRF1 or methylated eRF1 (0.05×) and eRF3Δ248 (0.5×) alone, omitting eIF5A and Rli1 (Figure 7C). All reactions were incubated at 30 °C for up to 120 min. The relative release activity was evaluated as described in Section 3.4.

### 3.7 Statistical analysis

Data are presented as mean ± standard deviation (SD) from three independent experiments (n = 3). Statistical significance between two groups was assessed using a two-tailed Welch’s t-test. P values are indicated in the figures.

## 4 Anticipated results

### 4.1 Preparation of GGQ-methylated eRF1

When *E. coli* Rosetta(DE3)pLysS was transformed with the co-expression vector eRF3Δ248_eRF1H6_Trm112_Mtq2/pET29b (Figure 2A) and cultured at large scale, His6-tagged eRF1 was successfully expressed and purified by Ni-NTA affinity chromatography followed by anion-exchange chromatography (HiTrap-Q), yielding approximately 9.45 mg of methylated eRF1 per 3 L culture with nearly homogeneous purity.

The methylation efficiency of the purified eRF1 was confirmed by MALDI-TOF mass spectrometry. The mass spectrum of the tryptic peptide containing the GGQ motif showed an ion peak corresponding to the monomethylated form, with no detectable peak corresponding to the unmethylated form, confirming that eRF1 was methylated at nearly 100% efficiency (Figure 2B).

### 4.2 Formation of pre-termination complex (PreTC) using the yPURE system

To examine PreTC formation in the yPURE system, translation of nLuc mRNA (Figure 3A) was analyzed in the presence or absence of release factors (eRFs; eRF1 and eRF3) (Figure 3B). Translation of nLuc mRNA without release factors gave only minimal nLuc luminescence activity (Figure 3B, eRFs(−), RNase(−)), while translation with release factors yielded substantial nLuc activity at 60 min (Figure 3B, eRFs(+), RNase(−)). RNase A treatment of translation products at 60 min gave comparable nLuc activities regardless of the presence or absence of release factors (Figure 3B, eRFs(+), RNase(+) and eRFs(−), RNase(+)), indicating that nLuc-PreTC was stably formed by simply omitting release factors from the yPURE system. Under these conditions, release factors partially released nLuc from approximately 30% of all PreTCs within 60 min.

### 4.3 Analysis of release factor activities

When methylated eRF1 was added to nLuc-PreTC at increasing concentrations, peptide release was induced in a concentration-dependent manner (Figure 4A). At the highest concentration tested (1 ×), the relative release activity reached approximately 20%. Notably, at a limiting concentration of eRF1 (0.05×), no detectable release activity was observed, indicating that this concentration is insufficient for eRF1 to act alone. Preliminary experiments showed that unmodified eRF1 yielded equivalent results under these conditions, indicating that GGQ methylation did not detectably affect peptide release activity in this assay. (See also Section 4.4; Figure 7 for a systematic comparison between unmodified eRF1 and methylated eRF1.) Therefore methylated eRF1 was used in the following experiments unless otherwise specified.

The addition of eRF3 to reactions containing this limiting concentration of eRF1 (0.05×) markedly stimulated peptide release in a concentration-dependent manner (Figure 4B). Here, full-length eRF3 and two N-terminally truncated variants were tested: eRF3Δ165, lacking the first 165 amino acid residues, and eRF3Δ248, lacking the first 248 amino acid residues, retaining only the translational GTPase domain homologous to eEF1A. All three eRF3 variants stimulated eRF1-mediated peptide release to comparable maximal levels of approximately 20% at saturating concentrations (0.5×), consistent with previous findings that the N-terminal domain of eRF3 is dispensable for peptide release stimulation (Shoemaker and Green, 2011). Notably, the maximal release activity achieved by eRF1 and eRF3 together did not exceed that of eRF1 alone at saturating concentrations (∼20%), consistent with the established role of eRF3 in accelerating the rate of eRF1 recruitment to the ribosome rather than enhancing the intrinsic peptide release efficiency.

eIF5A is known to stabilize the 3′-CCA end of peptidyl-tRNA and promote peptidyl transfer and peptide release reactions, and its hypusine modification is thought to enhance this activity by directly contacting the 3′-CCA end (Schuller et al., 2017; Schmidt et al., 2016). In the yPURE system, eIF5A at 1× was previously shown to fully rescue translational stalling caused by polyproline sequences (Abe et al., 2020b). Consistent with this, the addition of eIF5A (1×) to reactions containing eRF1 (0.05×) and eRF3Δ248 (0.5×) further stimulated peptide release beyond the ∼20% ceiling observed with eRF1 and eRF3 alone. Both eIF5A_Lys and eIF5A_Hyp promoted peptide release, and hypusine-modified eIF5A (eIF5A_Hyp) showed higher stimulatory activity than unmodified eIF5A (eIF5A_Lys). In the presence of saturating amounts of eIF5A_Hyp, the relative release activity reached approximately 50% (Figure 5).

Rli1 (ABCE1 in mammals) is an ATPase belonging to the E subfamily of the ABC protein superfamily that plays dual roles in translation termination and ribosome recycling (Shoemaker and Green, 2011). In termination, Rli1 binds to eRF1 after eRF3 dissociation from the pre-termination complex and stimulates peptide release; this contribution to termination has been shown to be independent of ATP hydrolysis. Subsequently, Rli1 promotes ribosome recycling by inducing dissociation of the large and small ribosomal subunits through ATP binding and hydrolysis. Consistent with the role of Rli1 in stimulating termination, the addition of Rli1 to reactions containing eRF1 (0.05×), eRF3Δ248 (0.5×), and eIF5A_Hyp (1×) further stimulated peptide release in a concentration-dependent manner, and the relative release activity reached approximately 80% at the highest concentration tested (2.5×) (Figure 6).

### 4.4 Context-dependent analysis of translation termination

To examine whether the identity of the penultimate amino acid of a protein affects peptide release, and whether eRF1 GGQ methylation influences this context-dependent effect, nLuc-PreTCs carrying a C-terminal alanine (Ala, GCA) or proline (Pro) residue specified by one of four codons (CCA, CCU, CCC, or CCG) (Figure 7A) were subjected to peptide release reactions under two conditions using unmodified eRF1 or methylated eRF1 (0.05×): a full release factor condition containing eRF3Δ248 (0.5×), eIF5A_Hyp (1×), and Rli1 (1×) (Figure 7B), and a minimal condition containing eRF3Δ248 (0.5×) alone, omitting eIF5A and Rli1 (Figure 7C). The two conditions were chosen to provide different release kinetics, allowing assessment of whether any context-dependent effects might be obscured by rapid release under the full release factor condition. The minimal condition yielded slower release kinetics for both unmodified and methylated eRF1, with peptide release reaching a plateau at approximately 60 min (Figure 7C) rather than 30 min as in the former condition (Figure 7B), while the kinetics of RNase A-mediated release remained largely unchanged between the two conditions (Figures 7B,C, RNase).

For all five constructs tested under both conditions, unmodified eRF1 and methylated eRF1 induced peptide release with comparable kinetics and to comparable extents, indicating that, under the experimental conditions used in this study, GGQ methylation did not detectably affect peptide release activity.

The relative release activity, calculated as a percentage of nLuc activity obtained upon RNase A treatment, reached approximately 100% or higher for all five constructs under both conditions, indicating that peptide release proceeded efficiently regardless of the identity of the penultimate amino acid. In particular, no suppression of peptide release was observed for any of the Pro-containing constructs relative to Ala(GCA), even under the slower release minimal condition omitting eIF5A and Rli1 (Figure 7C). This result contrasts with that of the aforementioned lysate-based Termi-nLuc assay (Biziaev et al., 2025). No substantial difference in release efficiency was observed among the four Pro codons tested, indicating that the lack of suppression by C-terminal proline in this system was not attributable to the specific codon used. The reproducibility of these results across two reaction conditions with different release kinetics suggests that the absence of detectable context-dependent effects in this system is unlikely to be due to the release reaction proceeding too rapidly to resolve such differences. Note that the mRNA constructs used in Figure 7 carry a UGA stop codon and a 3′-terminal poly(A)36 sequence, which potentially slows termination kinetics and stabilizes PreTC to promote eRF1•eRF3-mediated peptide release, respectively; however, preliminary experiments using constructs carrying a UAA stop codon without a poly(A) tail yielded comparable results, suggesting that neither modification substantially altered the rate-limiting step of the reaction under the current conditions. A higher mRNA concentration of 5 µM was used for PreTC formation in this section, because Pro-containing mRNA constructs showed lower translation efficiency than the Ala(GCA) construct; at 5 μM, PreTC formation from all five constructs reached comparable levels.

### 4.5 Assay performance and considerations

When using the yPURE-based Termi-nLuc assay, several practical considerations should be kept in mind. First, pre-termination complexes with short peptides, such as those derived from HiBiT mRNA, showed substantial background release in the absence of release factors that was comparable to eRF1-induced release, in agreement with a previous report (Alkalaeva et al., 2006). Therefore, mRNA encoding a full-length protein such as nLuc is recommended, as the long nascent peptide chain ensures stable PreTC formation with minimal spontaneous release.

Second, when forming PreTC, in addition to eRF1 and eRF3, the recycling factors Rli1, Dom34, and Hbs1 were also omitted from the yPURE system, and incubation was kept within 30 min. Extended incubation in the presence of these recycling factors can result in dissociation of PreTC into a 60S-nascent chain ribosomal complex (60S-RNC) and a free 40S subunit; in such cases, RNase A treatment still releases nLuc, but peptide release reactions by eRFs are no longer observed. This consideration should be taken into account when establishing similar reconstituted translation termination assays.

## 5 Discussion

The yPURE-based Termi-nLuc assay established in this study offers several advantages for the analysis of eukaryotic translation termination. PreTC is stably formed simply by omitting release factors from the yPURE system with minimal spontaneous peptide release, nLuc luminescence provides an extremely sensitive readout for peptide release, and no sucrose density gradient purification of PreTC is required, enabling rapid analysis. The established system has enabled monitoring of peptide release reactions in a concentration-dependent manner for individual release factors. For instance, while eRF1 and eRF3 alone supported peptide release at efficiencies of up to approximately 20%, the combined addition of eIF5A and Rli1 further increased the relative release activity to nearly 100%. These observations underscore the importance of these accessory factors, in addition to eRF1 and eRF3, for converting PreTC into a release-competent state, possibly by promoting an optimal orientation of the peptidyl-tRNA in the ribosome. To enable direct biochemical analysis of GGQ-methylated eRF1—a modification that previous biochemical studies have been unable to examine due to reliance on unmodified recombinant eRF1—we also established an *E. coli* co-expression system in which eRF1, eRF3Δ248, Mtq2, and Trm112 are co-expressed, yielding fully GGQ-methylated eRF1.

It should be noted that the total amount of PreTC, as assessed by RNase A-mediated nLuc release, decreased after 30 min of incubation under the current assay conditions (Figures 4–7). This may reflect progressive dissociation of PreTC into 60S-nascent chain complexes (60S-RNCs) and free 40S subunits as described in Section 4.5, with the resulting 60S-RNCs potentially becoming inaccessible to RNase A through aggregation or other structural rearrangements.

Recent high-resolution cryo-EM structural analysis of bacterial PreTC complexed with a release factor (RF1) revealed that peptide release proceeds *via* tRNA-mediated self-hydrolysis, in which RF binding reorients the 2′-OH group of A76 to serve as the nucleophile, rather than *via* direct attack by a water molecule as previously proposed (Aleksandrova et al., 2025). In this updated model, the methylated GGQ glutamine is thought to enhance RF binding affinity and precisely position the GGQ motif to stabilize the reaction intermediate, a role considered particularly important for efficient release at codons preceding proline or glycine residues (Pierson et al., 2016). Despite these advances in understanding, biochemical characterization of eRF1 GGQ methylation in eukaryotes has been entirely lacking to date.

In this study, neither GGQ methylation of eRF1 nor the identity of the penultimate amino acid, including the C-terminal proline previously shown to suppress release in a lysate-based assay (Biziaev et al., 2025), detectably affected peptide release activity in our system. Both observations may reflect the absence, in the current yPURE system, of factor(s) that promote loading of the eRF1•eRF3•GTP ternary complex onto the ribosomal A site—a step (Step A) that is kinetically distinct from the subsequent conformational change in eRF1 coupled to GTP hydrolysis that leads to peptidyl-tRNA hydrolysis (Step B) (Alkalaeva et al., 2006; Salas-Marco and Bedwell, 2004). GGQ methylation and penultimate amino acid identity are both thought to primarily influence Step B; if Step A is substantially slower than Step B under our current conditions, differences in Step B efficiency may be masked within the timescale of the overall reaction. In mammals, the poly(A)-binding protein PABP directly interacts with eRF3 and has been shown to enhance eRF1•eRF3 loading onto the ribosome independently of poly(A)-tail binding (Hoshino et al., 1999; Ivanov et al., 2016). It is worth noting here that the lysate-based Termi-nLuc assay (Biziaev et al., 2025), in which C-terminal proline suppressed peptide release, was performed using mRNA carrying a poly(A) tail in the presence of PABP. Whether the yeast counterpart of PABP, Pab1, fulfills an equivalent role in translation termination remains to be established. Supplementing the yPURE system with Pab1, or identifying other factors that accelerate Step A, represents a possible direction for expanding the application of the assay.

Our results, together with the previous studies, highlight the complexity of context-dependent regulation of translation termination. For example, the same penultimate amino acid can have opposite effects on peptide release depending on the context of the nascent chain: a C-terminal proline residue reportedly suppresses release in the context of a long nascent peptide but stimulates release in the context of a short peptide (Biziaev et al., 2025), whereas no detectable effect was observed in our system. Such complexity is further compounded by the fact that the influence of PABP/Pab1 on termination efficiency itself likely depends on context, given that termination at upstream ORFs (uORFs) or premature termination codons (PTCs), which are positioned distant from the poly(A) tail, would be expected to differ from termination at canonical stop codons. Future studies combining multiple complementary approaches, including the yPURE-based Termi-nLuc assay established here, lysate-based Termi-nLuc assays, and *in vivo* readthrough assays, will be important for elucidating the regulatory mechanisms underlying these context-dependent effects. Notably, unlike the lysate-based Termi-nLuc assay, in which PreTC is purified away from elongation factors prior to the peptide release reaction, the yPURE-based assay is performed in the presence of competing factors for the stop codon, such as eEF1A and aminoacyl-tRNAs; this feature makes it particularly well suited for analyzing stop codon readthrough and frameshifting. Furthermore, the compatibility of the yPURE system with *in vitro*-transcribed tRNAs (iVTtRNAs) carrying defined nucleotide modifications enables direct and quantitative analysis of how specific tRNA and mRNA modifications influence these context-dependent processes. The yPURE-based Termi-nLuc assay established in this study represents a versatile and high-throughput-compatible platform, with broad applicability not only for basic mechanistic studies of translation termination, but also for future screening applications, including the identification of small molecules that modulate termination efficiency or promote readthrough of premature stop codons.

## Data Availability

The original contributions presented in the study are included in the article/supplementary material, further inquiries can be directed to the corresponding author.
